# Systematic analysis of genetic variants in cancer-testis genes identified two novel lung cancer susceptibility loci in Chinese population

**DOI:** 10.7150/jca.40002

**Published:** 2020-02-03

**Authors:** Zhihua Li, Jianwei Tang, Wei Wen, Weibing Wu, Jun Wang, Jing Xu, Yue Yu, Zhicheng He, Xianglong Pan, Haixing Wei, Yining Zhu, Shuo Hu, Jing Cao, Hongbing Shen, Jun Que, Wei Wang, Quan Zhu, Liang Chen

**Affiliations:** 1 Department of Thoracic Surgery, The First Affiliated Hospital of Nanjing Medical University, Nanjing, 210029, China.; 2 Department of Epidemiology, Center for Global Health, International Joint Research Center, School of Public Health, Nanjing Medical University, Nanjing, Jiangsu, China.; 3 Jiangsu Key Lab of Cancer Biomarkers, Prevention and Treatment, Jiangsu Collaborative Innovation Center of Cancer Personalized Medicine, Nanjing Medical University, Nanjing, 211166, China.

**Keywords:** cancer-testis genes, lung cancer susceptibility, single nucleotide polymorphisms, Chinese population, Sequenom platform

## Abstract

Cancer-testis (CT) genes played important roles in the progression of malignant tumors and were recognized as promising therapeutic targets. However, the roles of genetic variants in CT genes in lung cancer susceptibility have not been well depicted. This study aimed to evaluate the associations between genetic variants in CT genes and lung cancer risk in Chinese population. A total of 22,556 qualified SNPs from 268 lung cancer associated CT genes were initially evaluated based on our previous lung cancer GWAS (Genome-wide association studies) with 2,331 cases and 3,077 controls. As a result, 17 candidate SNPs were further genotyped in 1,056 cases and 1,053 controls using Sequenom platform. Two variants (rs6941653, *OPRM1*, T > C, screening: OR = 1.24, 95%CI: 1.12-1.38, *P* = 2.40×10^-5^; validation: OR = 1.18, 95%CI: 1.01-1.37, *P* = 0.039 and rs402969, *NLRP8*, C > T, screening: OR = 1.15, 95%CI: 1.04-1.26, *P* = 0.006; validation: OR = 1.16, 95%CI: 1.02-1.33, *P* = 0.028) were identified as novel lung cancer susceptibility variants. Stratification analysis indicated that the effect of rs6941653 was stronger in lung squamous cell carcinoma (OR = 1.36) than that in lung adenocarcinoma (OR = 1.15,* I^2^* = 77%, *P* = 0.04). Finally, functional annotations, differential gene expression analysis, pathway and gene ontology analyses were performed to suggest the potential functions of our identified variants and genes. In conclusion, this study identified two novel lung cancer risk variants in Chinese population and provided deeper insight into the roles of CT genes in lung tumorigenesis.

## Introduction

Lung cancer has been the most frequently diagnosed caner type and the leading cause of cancer-related deaths for decades in China 1. The tumorigenesis of lung cancer was a multiple-stage process, and both environmental and genetic factors were involved. It was estimated that the heritability of lung cancer was about 15.2% in Chinese population 2. However, up till now, GWAS (Genome-Wide Association Study)-reported lung cancer associated single nucleotide polymorphisms (SNPs) could only account for limited lung cancer heritability (less than 1%) 2, 3. Therefore, more effective strategies were wanted to identify novel lung cancer risk loci based on GWAS data.

Epigenetic alterations have been recognized as an important feature of tumorigenesis 4, 5. Notably, cancer-testis (CT) genes, which were restrictedly expressed in germ cells and malignant tumor cells, were usually activated through epigenetic mechanisms 6. The activation of CT genes in cancer samples made them oncogene candidates and their remained high immunogenicity made them perfect immunotherapeutic targets for cancer treatment 7, 8. In addition, associations between genetic variants in CT genes and the susceptibility of cancers have been described in previous studies. For example, genetic variants in *HORMAD2* and *GPATCH2* were reported associated with lung cancer risk 9, 10, and variants in *CTNNA2*, *CCDC33* and *SPAG17* showed significant association with the susceptibility of breast cancer 11, 12. All these studies suggested that genetic variants in CT genes could also contribute to the development of cancers.

Therefore, systematic analysis of the associations between genetic variants in CT genes and lung cancer risk could help identify more novel lung cancer susceptibility loci. In our previous study, we performed a systematic identification of CT genes in 19 cancer types based on multiple public-available databases 13. As a result, 876 novel CT genes in 19 cancer types were recognized. In lung cancer, we identified 268 CT genes (including 61 known CT genes) that were activated in at least 2% of cancer samples 13. This finding provided us an unprecedented opportunity to explore the associations between genetic variants in CT genes and the susceptibility of lung cancer.

In this study, a two-stage case control study was performed. The NJMU GWAS, which has been established in our previous study, was used to screen candidate lung cancer risk variants 9. These promising variants were further validated in an independent Chinese population with a total of 1,056 lung cancer cases and 1,053 controls based on the Sequenom MassARRAY iPLEX platform. This study would identify novel lung cancer susceptibility loci in Chinese population and help reveal the roles of genetic variants in CT genes in the development of lung cancer.

## Materials and Methods

### Study subjects

Two independent datasets were used in this study. The NJMU GWAS contained 2,331 lung cancer cases and 3,077 controls, and was used as screening dataset. The detailed information about the study subjects in NJMU GWAS was described previously 9. Genotype imputation for NJMU GWAS was performed using IMPUTE2 and Shapeit v2 with the 1000 Genomes Project (the Phase III integrated variant set release, across 2504 samples) as the reference 14. The validation dataset consisted of 1,056 lung cancer cases and 1,053 controls. All the cases were histopathologically confirmed patients and were recruited from the First Affiliated Hospital of Nanjing Medical University and Jiangsu Cancer Hospital. Controls were obtained from a screening program for non-infectious diseases conducted in Jiangsu Province and matched to the cases for age and gender. All the participants have signed the informed consent acknowledgement and this study was approved by the ethical review board of Nanjing Medical University.

### Screening for candidate risk variants in CT genes

**Figure 1:**
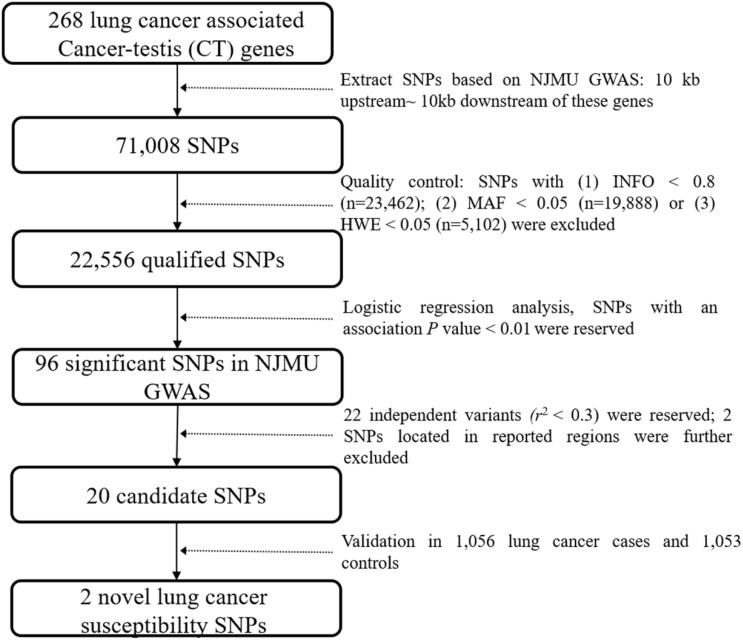
Flowchart of this study.

As shown in **Figure 1**, genetic variants in 268 lung cancer associated CT genes (**Supplementary Table 1**, including the 10 kb upstream and downstream of these genes) were extracted based on NJMU GWAS. Initially, 71,008 SNPs were reserved. Further, SNPs satisfied any of the following criteria were excluded: (1) imputation quality score INFO < 0.8; (2) minor allele frequency (MAF) < 0.05; (3) hardy-weinberg equilibrium (HWE) < 0.05. This resulted in 22,556 qualified SNPs reserved for association analysis. Among them, 96 SNPs showed significant association (*P* < 0.01) with lung cancer risk in NJMU GWAS. Furthermore, SNPs that were located in previously reported regions or showed a high correlation (*r*^2^ > 0.3) with each other (SNPs with the least *P* value were reserved) were excluded. Finally, 20 independent SNPs were retained as lung cancer risk variant candidates.

### Variants validation using Sequenom platform

In this study, genotyping in the validation stage was performed using the Sequenom MassARRAY iPLEX platform (Sequenom Inc. San Diego, CA, USA) according to the protocol 15, 16. Primers for 17 out of 20 candidate SNPs were successfully constructed. In particular, rs144031443 and rs150492976 were replaced by rs75932085 (*r*^2^ = 0.66, Chinese Han population) and rs4726004 (*r*^2^ = 1), respectively. The genotyping experiment was performed by technicians who were blinded to the status of case or control. Cases and controls were mixed in each 384-well plate with two water samples as blank controls. In addition, 5% of samples were randomly selected for repeat genotyping, which yielded a concordance rate of 100%. Primer sequences used in this study were shown in **Supplementary Table 2**.

### Functional annotations, pathway and Gene Ontology (GO) analyses

To gain insight into the potential functions of our identified SNPs and their related variants (*r*^2^ ≥ 0.6), several public-available databases, including HaploReg v4.1 (https://pubs.broadinstitute.org/mammals/haploreg/), ENCODE, rSNPBase (http://rsnp.psych.ac.cn/index.do) 17, 18, SNP2TFBS (https://ccg.epfl.ch/snp2tfbs/) 19, PINES (http://genetics.bwh.harvard.edu/pines/index.html) and RegulomeDB (http://regulome.stanford.edu/) 20, were used for functional annotations. Pathway enrichment and GO analyses were performed based on DAVID Bioinformatics Resources 6.8 (https://david.ncifcrf.gov/home.jsp) 21.

### Statistical analysis

The chi-square test was used for the comparison of categorical variables between cases and controls, while the Student's t test was adopted for continuous variables. Associations between genetic variants and lung cancer risk were evaluated (Odd Ratios, OR and 95% confidence intervals, 95%CI) using logistical regression analysis. Age, sex, smoking status and principal components were adjusted. The differential gene expression analysis was conducted using RNA sequence data (107 paired lung cancer and adjacent normal tissues) from the Cancer Genome Atlas (TCGA) database, and was further validated in 60 pairs of lung tumor and normal tissues from GSE19804. The expression quantitative trait loci (eQTL) analysis was performed based on the GTEx v7 database (383 lung tissues).* OPRM1* and *NLRP8* co-expressed genes were screened based on lung cancer RNA-seq (TCGA) using Pearson correlation analysis. All the analyses were performed using R (3.5.2) or Plink 1.9.

## Results

### Characteristics of study subjects

The characteristics of study subjects were shown in **Table 1**. In brief, a total of 3,387 lung cancer cases and 4,130 controls were included in this study. Lung adenocarcinoma (LUAD) was the most common histological type and accounted for more than 50% of all the lung cancer cases. Lung squamous cell carcinoma (SCC) accounted for 35.3% and 21.8% in NJMU GWAS and validation samples, respectively. The distribution of age, sex and smoking status was comparable between lung cancer cases and controls in validation samples (*P* > 0.05).

### Two novel lung cancer susceptibility variants

As mentioned above, 17 of 20 lung cancer candidate SNPs were successfully genotyped in validation stage. Associations between 17 candidate variants and lung cancer risk were shown in **Table 2**. Notably, only rs6941653 and rs402969 were significant in both screening (rs6941653, *OPRM1*, T > C, OR = 1.24, 95%CI: 1.12-1.38, *P* = 2.40×10^-5^; rs402969, *NLRP8*, C > T, OR = 1.15, 95%CI: 1.04-1.26, *P* = 0.006) and validation datasets (rs6941653, *OPRM1*, T > C, OR = 1.18, 95%CI: 1.01-1.37, *P* = 0.039; rs402969, *NLRP8*, C > T, OR = 1.16, 95%CI: 1.02-1.33, *P* = 0.028). Therefore, rs6941653 and rs402969 were considered as novel lung cancer risk variants in Chinese population.

### Stratification analysis and interaction analysis

**Figure 2:**
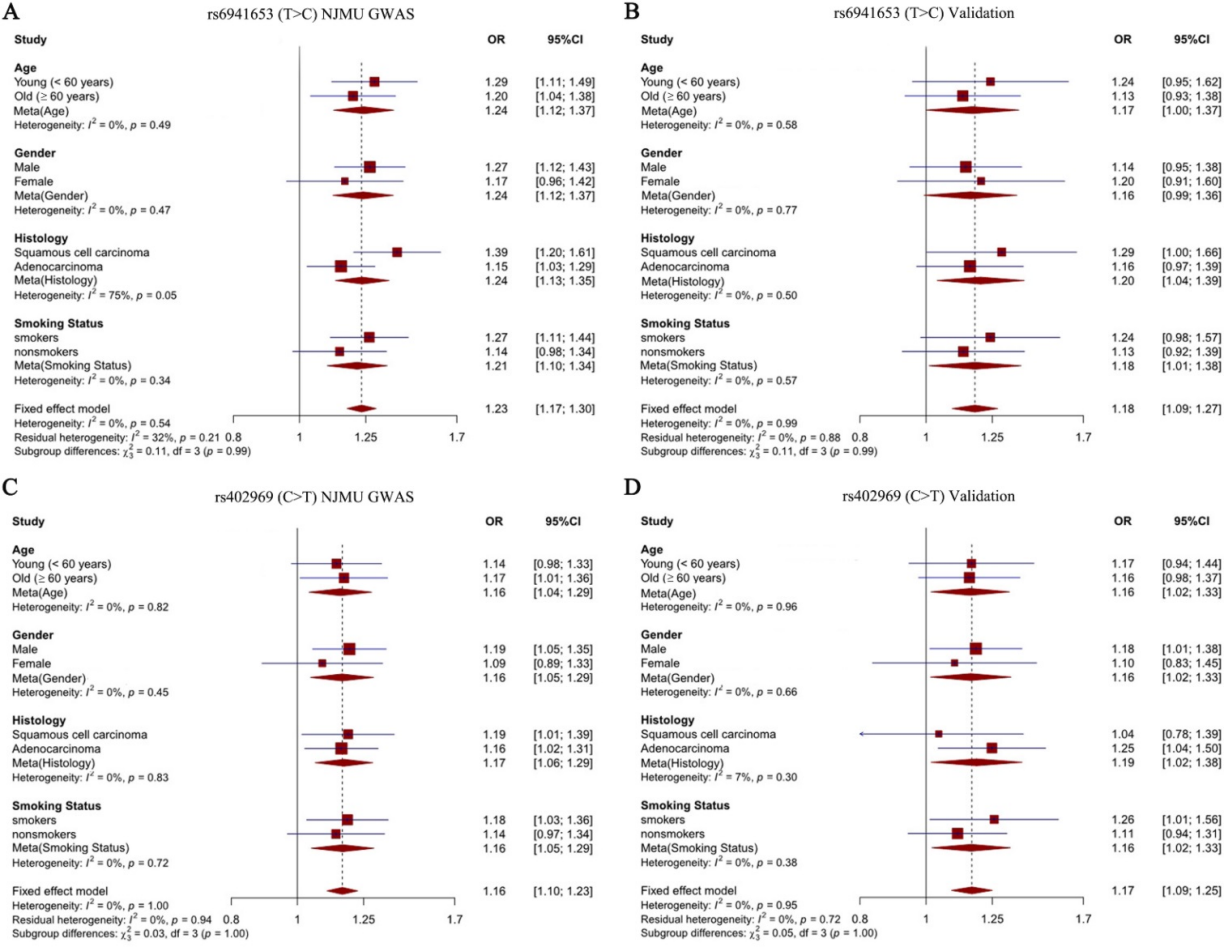
** Forest plot of rs6941653 and rs402969 in each subgroup. (A)** Forest plot of rs6941653 in NJMU GWAS; **(B)** Forest plot of rs6941653 in validation dataset; **(C)** Forest plot of rs402969 in NJMU GWAS; **(D)** Forest plot of rs402969 in validation samples.

**Figure 3:**
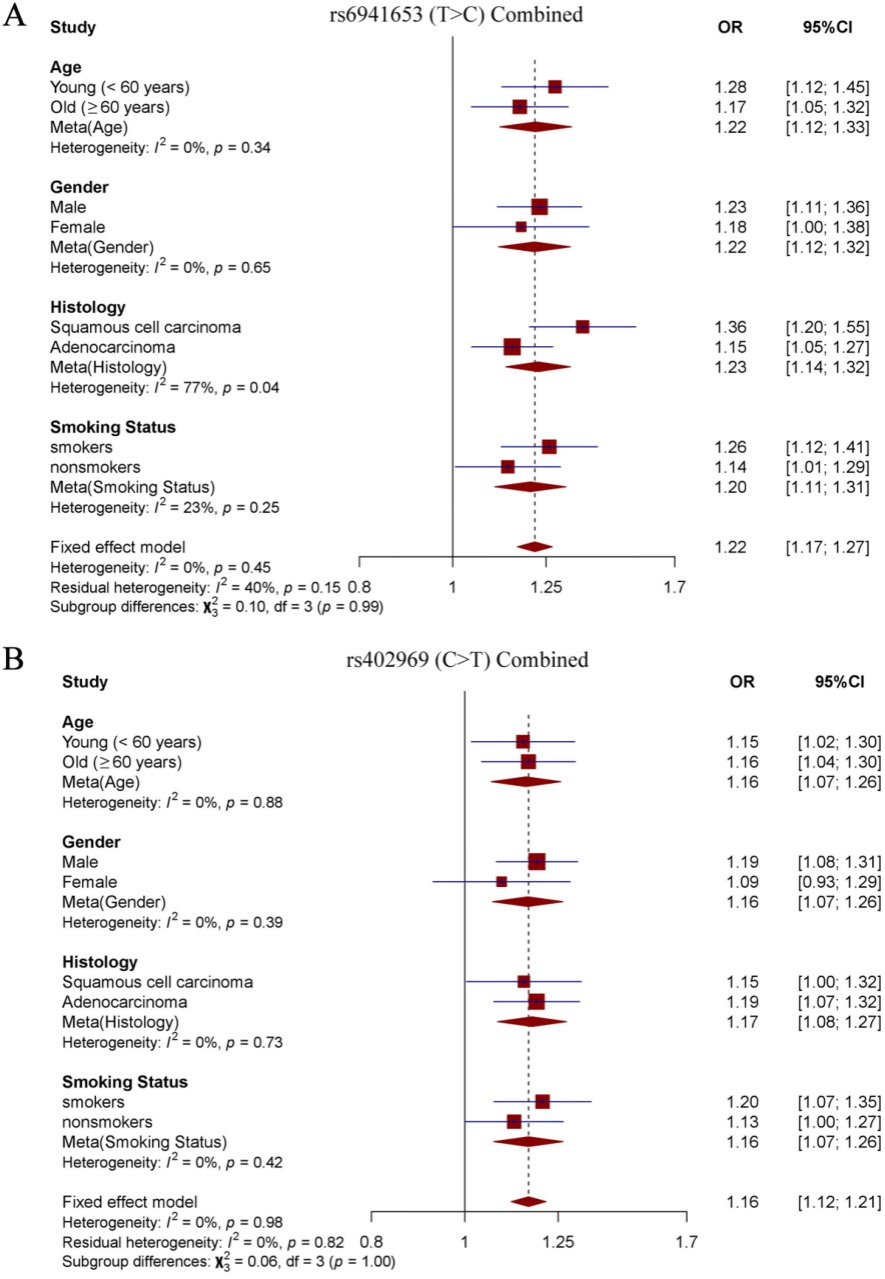
** Forest plot of rs6941653 and rs402969 in combined datasets. (A)** Forest plot of rs6941653 in combined data; **(B)** Forest plot of rs402969 in combined dataset.

To explore the effects of our identified two SNPs in different subgroup populations, stratification analysis was further performed. Study subjects were divided into four subgroups according to age (young < 60 years old vs old ≥ 60 years old), sex (males vs females), smoking status (smokers vs nonsmokers) and histological subtypes (SCC vs LUAD). Notably, we observed a significantly stronger effect of rs6941653 in SCC (NJMU GWAS: OR = 1.39; Validation: OR = 1.29; Combined: OR = 1.36) than that in LUAD (NJMU GWAS: OR = 1.15; Validation: OR = 1.16; Combined: OR = 1.15, *I^2^* = 77%, *P* = 0.04,** Figure 2A-2B**, **Figure 3A**). In contrast to rs6941653, SNP rs402969 showed a similar effect on lung cancer risk in various subgroup populations (**Figure 2C-D**, **Figure 3B**). In addition, interactions between our identified lung cancer risk variants (rs6941653 and rs402969) and smoking were evaluated. However, no significant interaction was found (**Supplementary Table 3**,* P* for interaction > 0.05). Similarly, there was no significant interaction between variant rs6941653 and rs402969 (**Supplementary Table 4**,* P* for interaction > 0.05).

### Functional annotations and eQTL analysis

In order to suggest the potential functions of our identified two novel variants and their related SNPs, functional annotations were performed. As shown in **Table 3**, rs6941653 was not located in regulatory element regions, such as promoter, enhancer, transcription factor (TF) binding sites or DNase peak. Strikingly, rs9397692 (*r*^2^ = 0.70 with rs6941653) was an eQTL and could influence the binding of transcription factor NFATC2. What's more, rs9397692 had a Regulome DB score of 4, suggesting that it was located in TF binding site and DNase peak. All these results indicated that rs9397692 might be the functional variant, which could affect the binding of specific transcription factor NFATC2. In the second risk loci, rs805165 (*r*^2^ = 0.90 with rs402969) was predicted to be an eQTL and could modify the affinity to TF BRCA1. Consistently, the Regulome DB score of rs805165 was 5 (TF binding or DNase peak). Taken together, rs805165 might be the functional variant in the second loci. Furthermore, eQTL analysis in lung tissues was conducted based on GTEx v7 database. Unfortunately, the expression of *OPRM1* and *NLRP8* in lung tissues was not sufficient for eQTL calculation. Therefore, associations between our identified variants and the expression of their host genes (*OPRM1* and *NLRP8*) were not evaluated. Notably, rs805165 was found to be significantly associated with the expression of AC010525.7 and AC024580.1 (**Supplementary Figure 1**). However, the functions of AC010525.7 and AC024580.1 have not been reported in previous studies.

### Differential gene expression, pathway and GO analyses

**Figure 4:**
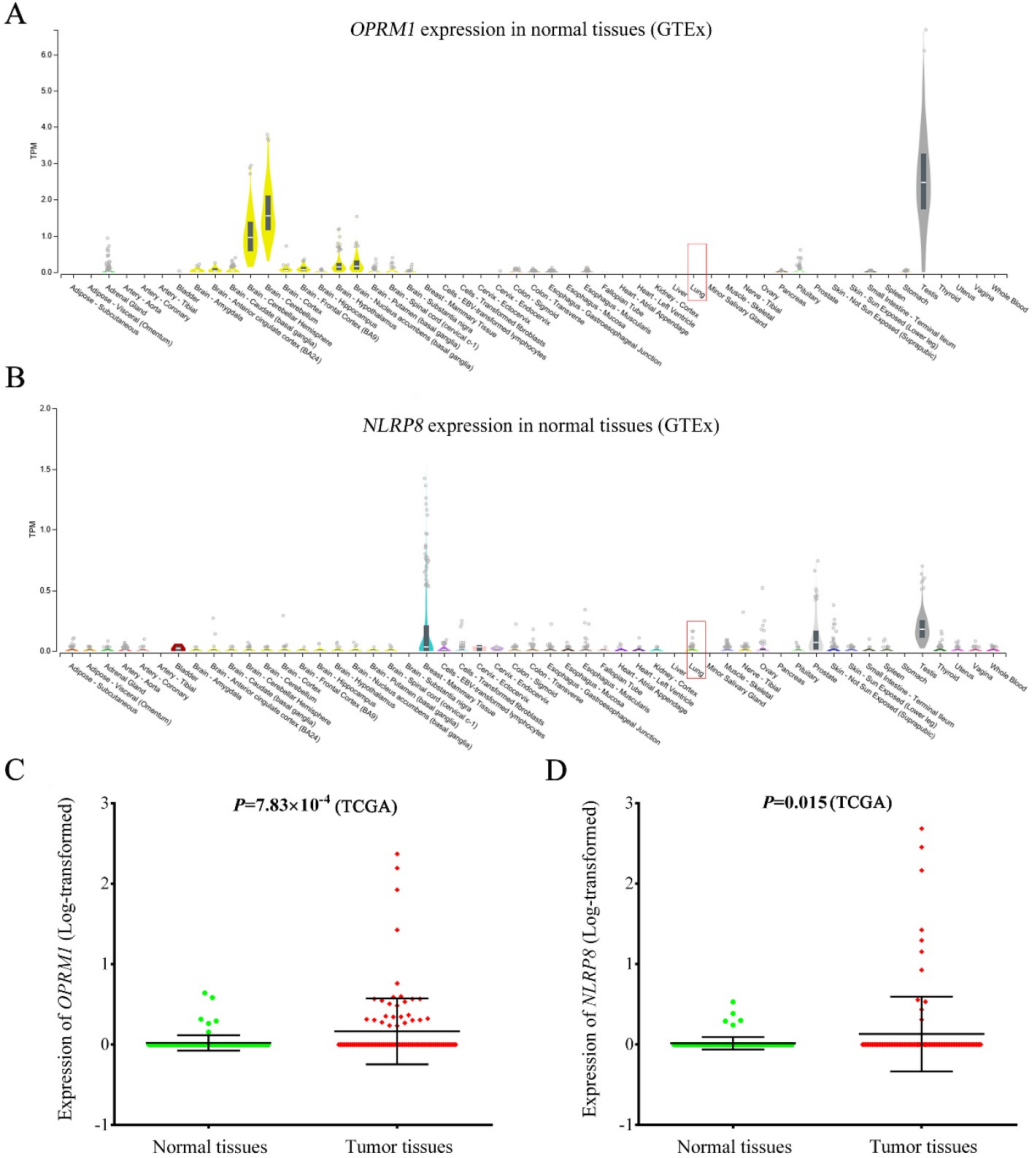
** Expression of *OPRM1* and *NLRP8* in normal lung tissues and lung tumor tissues.** *OPRM1* **(A)** and *NLRP8* **(B)** expression in a variety of normal tissues based on GTEx database. Lung tissue was marked in red box; The expression of *OPRM1* **(C)** and *NLRP8* **(D)** in 107 pairs of lung cancer tissues and adjacent normal tissues based on TCGA database.

As shown in **Figure 4**, *OPRM1* was not expressed in normal lung tissues (GTEx database), but showed abundant expression in testis and brain tissues (**Figure 4A**). The consistent finding was observed in lung cancer adjacent normal tissues based TCGA database. However, the expression of *OPRM1* was significantly elevated in lung tumor tissues compared to that in normal tissues (*P* = 7.83 × 10^-4^,** Figure 4C**). Similarly, *NLRP8* expression was aberrantly upregulated in tumor tissues (**Figure 4D**, *P* = 0.015). These findings were further validated in 60 paired lung tumor tissues and adjacent normal tissues from GSE19804 (**Supplementary Figure 2**). Pathway enrichment analysis showed that *OPRM1* co-expressed genes (Fold enrichment: 14.0, *P* = 2.10 × 10^-214^) and *NLRP8* co-expressed genes (Fold enrichment: 6.7, *P* = 3.10 × 10^-110^, **Supplementary Table 5**) were enriched in the olfactory transduction pathway. The GO analysis indicated that *OPRM1* co-expressed genes might participate in various biological processes, such as immune response, signaling pathway and cell differentiation. The co-expressed genes with *NLRP8* could take part in the sensory perception, keratinocyte differentiation and G-protein coupled receptor signaling pathway.

## Discussion

Although lung cancer GWASs have identified dozens of lung cancer susceptibility loci, more lung cancer associated variants remained to be identified 3. Cancer-testis genes were recognized as promising therapeutic targets for cancers due to their high immunogenicity in tumor tissues 22, 23. However, the roles of genetic variants in CT genes in the development of lung cancer have not been revealed in previous studies.

In the current study, a two-stage case-control study was performed to systematically evaluate the associations between genetic variants in CT genes and the risk of lung cancer in the Chinese population. A total of 22,556 qualified SNPs from 268 lung cancer associated CT genes were initially analyzed and 17 candidate SNPs were genotyped using the Sequenom platform. Finally, two variants (rs6941653 in *OPRM1* and rs402969 in *NLRP8*) were identified as novel susceptibility loci of lung cancer in Chinese population.

SNP rs6941653 was located in the intron of *OPRM1* (opioid receptor mu 1, 6q25.2), which encoded one of the opioid receptors 24. Previous studies revealed that genetic variants in *OPRM1* could modulate the dependence to multiple drugs or chemical agents, including nicotine, cocaine, alcohol 25-27. Due to these properties, *OPRM1* has been reported associated with the progression of Alzheimer's disease, Parkinson's disease, Schizophrenia and so on 28-30. In lung cancer, Lennon FE *et al* found that *OPRM1* (also known as *MOR*) expression was elevated in several human non-small cell lung cancer cell lines. What's more, overexpression of *OPRM1* significantly promoted the proliferation of lung cancer *in vitro* and *in vivo* 31, 32. Consistently, *OPRM1* was recognized as a novel CT gene in lung cancer because of its aberrant expression in lung tumor tissues according to our previous study 13. In the current study, rs6941653 in *OPRM1* was further identified associated with lung cancer risk in Chinese population. Strikingly, stratification analysis indicated that rs6941653 showed a stronger effect in the SCC population. With regard to the potential mechanisms, we speculated that nicotine dependence induced by *OPRM1* might partially account for this. Functional annotations for rs6941653 and their LD variants suggested that rs9397692, which was in the DNase peak and could affect the binding of TF NFATC2, might be the functional variant in these loci.

The rs402969 was located in the upstream of *NLRP8*, which belonged to the member of the nucleotide-binding oligomerization domain/ leucine rich repeat/ pyrin domain containing (*NLRP*) subfamily 33. This gene family was involved in innate immunity, inflammasome formation and mammalian reproduction 34-36. However, the functions and roles of *NLRP8* in cancers have not been described in the previous study. In this study, *NLRP8* expression was found significantly upregulated in lung cancer tissues. What's more, rs805165 (LD with rs402969) was predicted in the binding sites of TF BRCA1 or DNase peak, suggesting a potential role as a regulator of gene expression. As expected, rs805165 showed a significant association with the expression of AC010525.7 and AC024580.1. Nevertheless, the functions of them have not been elucidated. Unfortunately, the expression of *NLRP8* in lung tissues was not sufficient for eQTL evaluation. Function studies were needed to reveal the role of *NLRP8* in lung cancer and identify the potential functional variants in these loci.

In conclusion, we performed a systematic evaluation of the associations between genetic variants in CT genes and the risk of lung cancer. Two novel lung cancer susceptibility loci were successfully identified in Chinese population. This study could improve our understanding of the roles of CT genes in the tumorigenesis of lung cancer. However, functional studies were wanted to verify the functional variants and reveal the potential mechanisms.

## Supplementary Material

Supplementary figures and tables.

## Figures and Tables

**Table 1:** Characteristics of subjects in screening and validation stages.

Characteristics	NJMU GWAS				Validation		
	Cases	Controls	*P* ^a^		Cases	Controls	*P* ^a^
**Sample size**	2331	3077			1056	1053	
**Gender**			<0.001				0.782
Male	1711(73.4%)	2086(67.8%)			700(66.3%)	704(66.9%)	
Female	620(26.6%)	991(32.2%)			356(33.7%)	349(33.1%)	
**Smoking**			<0.001				0.432
Yes	1506(64.6%)	1309(42.5%)			464(43.9%)	444(42.2%)	
No	825(35.4%)	1768(57.5%)			592(56.1%)	609(57.8%)	
**Age (years)**			0.124				0.317
Mean	60.05±10.27	60.47±9.66			61.40±9.59	60.98±9.95	
< 60	1111(47.7%)	1429(46.4%)			422(40.0%)	384(36.5%)	
≥ 60	1220(52.3%)	1648(53.6%)			634(60.0%)	669(63.5%)	
**Histology**							
Squamous cell carcinoma	822(35.3%)	/			230(21.8%)	/	
Adenocarcinoma	1304(55.9%)	/			622(58.9%)	/	
Other	205(8.8%)	/			204(19.3%)	/	

^a^ The chi-square test was used for gender, smoking and histology. Student's t test was adopted for age.

**Table 2:** Associations between 17 candidate SNPs and lung cancer risk in screening and validation datasets.

SNP	BP	CytoBand	Gene	Allele ^a^	NJMU GWAS			Validation			Combined	
					OR (95%CI) ^b^	*P* ^b^		OR (95%CI) ^c^	*P ^c^*		OR (95%CI)	*P*
**rs6941653**	**chr6:154531020**	**6q25.2**	***OPRM1***	**C/T**	**1.24(1.12,1.38)**	**2.40E-05**		**1.18(1.01-1.37)**	**0.039**		**1.22(1.12-1.33)**	**7.84E-06**
rs6851719	chr4:94120480	4q22.2	*GRID2*	C/A	1.18(1.08,1.29)	3.82E-04		1.05(0.92-1.20)	0.462		1.14(1.06-1.23)	6.15E-04
rs8139987	chr22:25131425	22q11.23	*PIWIL3*	T/G	1.28(1.12,1.47)	4.01E-04		0.92(0.76-1.12)	0.402		1.09(0.79-1.51)	0.588
rs79727953	chr7:102449418	7q22.1	*FBXL13*	A/C	1.24(1.10,1.39)	4.76E-04		1.22(0.98-1.45)	0.073		1.23(1.12-1.36)	1.53E-05
rs77027865	chr1:152863288	1q21.3	*SMCP*	G/A	1.34(1.12,1.59)	1.13E-03		1.14(0.88-1.46)	0.321		1.27(1.10-1.46)	8.12E-04
rs3123484	chr1:182884429	1q25.3	*SHCBP1L*	T/C	0.75(0.63,0.89)	1.34E-03		1.43(1.12-1.82)	0.002		1.03(0.55-1.94)	0.928
rs145033304	chr19:56514378	19q13.43	*NLRP5*	T/C	0.86(0.78,0.94)	1.37E-03		0.84(0.66-1.07)	0.169		0.86(0.79-0.93)	3.10E-04
rs12645087	chr4:178779663	4q34.3	*RP11-389E17.1*	T/C	0.80(0.69,0.92)	1.70E-03		1.02(0.85-1.23)	0.827		0.90(0.71-1.14)	0.364
rs9478496	chr6:154333183	6q25.2	*OPRM1*	C/T	1.30(1.10,1.54)	2.56E-03		0.96(0.75-1.22)	0.737		1.13(0.84-1.52)	0.415
rs144031443	chr4:94171881	4q22.2	*GRID2*	A/G	0.76(0.63,0.91)	2.70E-03		0.86(0.62-1.20)	0.376		0.78(0.67-0.92)	2.30E-03
rs17135666	chr16:1942405	16p13.3	*MEIOB*	T/C	0.79(0.68,0.92)	2.96E-03		1.05(0.84-1.31)	0.669		0.90(0.68-1.19)	0.458
rs7546603	chr1:182529584	1q25.3	*RGSL1*	C/T	1.20(1.06,1.35)	4.50E-03		1.03(0.86-1.23)	0.747		1.15(1.04-1.26)	6.76E-03
rs79461429	chr4:93761350	4q22.2	*GRID2*	G/A	1.18(1.05,1.32)	5.94E-03		1.14(0.97-1.37)	0.118		1.17(1.06-1.29)	1.38E-03
**rs402969**	**chr19:56450708**	**19q13.43**	***NLRP8***	**T/C**	**1.15(1.04-1.26)**	**6.00E-03**		**1.16(1.02-1.33)**	**0.028**		**1.15(1.07-1.24)**	**2.38E-04**
rs150492976	chr7:150875087	7q36.1	*GBX1*	T/C	0.86(0.78,0.96)	7.05E-03		0.99(0.85-1.15)	0.855		0.90(0.83-0.99)	0.025
rs175150	chr22:17311027	22q11.1	*XKR3*	A/C	1.14(1.04,1.26)	7.96E-03		1.00(0.87-1.14)	0.960		1.09(1.00-1.18)	0.041
rs60813831	chr19:43930119	19q13.31	*TEX101*	C/G	0.89(0.82,0.97)	8.12E-03		0.93(0.83-1.05)	0.246		0.90(0.84-0.97)	4.48E-03

a: Minor/Major allele; b: Age, gender, smoking pack-years and PCA were adjusted; c: Age, gender and smoking status were adjusted. SNPs that showed consistent association results and had an association *P* value < 0.05 in both NJMU GWAS and validation datasets were marked in bold.

**Table 3:** Functional annotations for our identified SNPs and their related variants (*r*^2^ ≥ 0.6).

r^2^	SNP	Motifs	PINES^ a^	Regulome DB ^b^	Regulation ^c^	eQTL ^d^	SNP2TFBS ^e^
0.75	rs10485060	HNF4, Pax-4, RXRA	0.433	6	no	yes	HNF4G, HNF4A, SOX10
0.80	rs2272381	Foxp1, Hoxd8, Pou3f2	0.297	6	yes	yes	/
0.95	rs2293537	7 altered motifs	0.473	No Data	yes	no	Crx
0.65	rs35184807	AP-1	3'-UTR	5	yes	no	/
0.77	rs60145555	AP-4, ELF1	0.178	5	no	no	/
0.95	rs61307239	EBF, ZEB1	0.372	6	no	no	/
0.70	rs62434770	5 altered motifs	0.281	No Data	no	no	Pdx1, Prrx2
0.68	rs62436491	Foxd3, Foxj1	0.868	5	no	no	/
0.92	rs6900677	Myf	0.379	6	no	no	/
0.81	rs6921548	6 altered motifs	0.048	5	yes	no	/
1.00	rs6941653	Maf, PTF1-beta	0.485	No Data	no	no	/
0.70	rs72574410	4 altered motifs	0.232	6	no	no	SPIB
0.75	rs74439078	5 altered motifs	0.414	6	no	no	/
0.73	rs9371779	BDP1, E2F	0.337	6	no	no	/
0.95	rs9383694	9 altered motifs	0.438	5	yes	no	/
0.94	rs9383695	BATF, Irf, SP1	0.367	No Data	yes	no	/
0.78	rs9384190	5 altered motifs	0.408	6	no	yes	RUNX1, RUNX2
0.75	rs9397179	Pou2f2	0.369	6	no	no	/
0.70	rs9397692	NF-AT, NF-AT1	0.086	4	no	yes	NFATC2
0.94	rs9397696	6 altered motifs	0.601	No Data	no	no	Klf4, Klf1
0.74	rs9478516	10 altered motifs	0.498	6	no	no	NFATC2
0.74	rs9478517	DMRT5, Foxa	0.498	6	no	no	Sox5
0.91	rs9479791	AP-1	0.424	No Data	no	yes	/
0.87	rs451276	11 altered motifs	0.621	No Data	no	no	Klf4, Klf1
1.00	rs402969	Hbp1, TCF12, ZBRK1	0.512	5	no	no	/
0.90	rs395589	10 altered motifs	0.512	6	no	no	/
0.92	rs381249	SIX5	0.512	6	no	no	/
0.90	rs448020	Evi-1, PEBP, RXRA	0.512	5	no	no	/
0.86	rs370095	4 altered motifs	0.668	No Data	no	no	/
0.90	rs371382	5 altered motifs	0.396	No Data	no	no	Pax2
0.90	rs805166	5 altered motifs	0.408	No Data	no	no	ZNF263
0.90	rs809275	Ets, TBX5, YY1	0.408	3a	yes	no	/
0.90	rs805165	BHLHE40, BRCA1, Maf	0.475	5	no	yes	BRCA1
0.69	rs809800	GR, STAT	0.475	6	no	no	/
0.90	rs805164	6 altered motifs	0.475	No Data	no	no	SP1
0.68	rs413691	/	0.483	6	yes	no	MEF2C, MEF2A
0.90	rs429498	6 altered motifs	0.483	No Data	no	no	SP2
0.82	rs409402	5 altered motifs	0.68	5	yes	no	/
0.81	rs393535	NRSF, Zfx	0.68	No Data	yes	no	Zfx
0.90	rs810903	STAT	0.672	No Data	no	yes	/
0.85	rs306508	4 altered motifs	0.406	No Data	no	yes	/
0.85	rs306507	/	missense	No Data	no	yes	/
0.85	rs306506	8 altered motifs	missense	No Data	no	no	BRCA1
0.85	rs7343161	Irf, SIX5, STAT	0.623	5	no	yes	/
0.85	rs306502	Cdx	0.685	No Data	no	no	/
0.85	rs306501	5 altered motifs	0.763	6	no	yes	/

a: PINES (http://genetics.bwh.harvard.edu/pines/index.html) provided a powerful in silico method to prioritize functional variants. SNPs with lower* P* values were more likely to be functional variants. b: Scores for regulatory elements based on RegulomeDB website (http://regulome.stanford.edu/); “3a”: TF binding + any motif + DNase peak; “4”: TF binding + DNase peak; “5”: TF binding or DNase peak; “6”: other. c: Proximal regulation or Distal regulation based on rSNPBase database (http://rsnp.psych.ac.cn/index.do); d: eQTL with experimental evidence based on rSNPBase database. e: The potential transcription factors were predicted based on SNP2TFBS (https://ccg.epfl.ch/snp2tfbs/).

## Abbreviations

CT: cancer-testis
GWAS: Genome-wide association studies
SNP: single nucleotide polymorphisms
MAF: minor allele frequency
HWE: hardy-weinberg equilibrium
GO: Gene Ontology
OR: Odd Ratios
CI: confidence intervals
eQTL: expression quantitative trait loci
TCGA: The Cancer Genome Atlas
SCC: squamous cell carcinoma
LUAD: lung adenocarcinoma
TF: transcription factor
