# Supplementary material for: Systematic analysis of genetic variants in cancer-testis genes identified two novel lung cancer susceptibility loci in Chinese population
Source: J Cancer. 2020 Feb 3;11(7):1985–93. doi: 10.7150/jca.40002 (PMC7052880; doi:10.7150/jca.40002)
Supplement: Supplementary file 1 — Supplementary figures and tables. [file jcav11p1985s1.pdf]

Table S1. The characteristics of 268 CT genes identified in our previous study.

ENname	CHR	Start	End	Strand	Gene	Known
ENSG00000007350	chrX	153524024	153558700	+	TKTL1	NO
ENSG00000046774	chrX	141290131	141293076	-	MAGEC2	YES
ENSG00000068985	chrX	49452053	49460596	-	PAGE1	YES
ENSG00000070886	chr1	22890057	22930087	+	EPHA8	NO
ENSG00000075886	chr2	132233666	132240507	+	TUBA3D	NO
ENSG00000080572	chrX	106449862	106487473	+	PIH1D3	NO
ENSG00000095627	chr10	115939029	115992063	+	TDRD1	YES
ENSG00000099399	chrX	30233677	30238206	+	MAGEB2	YES
ENSG00000101448	chr20	44165625	44176391	-	EPPIN	YES
ENSG00000101890	chrX	108616135	108725301	-	GUCY2F	NO
ENSG00000102387	chrX	100523241	100548059	-	TAF7L	YES
ENSG00000103546	chr16	55689516	55740104	+	SLC6A2	NO
ENSG00000104755	chr8	39601254	39695808	-	ADAM2	YES
ENSG00000104818	chr19	49535169	49536495	+	CGB2	NO
ENSG00000104941	chr19	46298968	46318577	-	RSPH6A	NO
ENSG00000106410	chr7	144094333	144107320	-	NOBOX	NO
ENSG00000109832	chr11	125773271	125793158	+	DDX25	NO
ENSG00000112038	chr6	154331631	154568001	+	OPRM1	NO
ENSG00000112238	chr6	100054606	100063454	+	PRDM13	NO
ENSG00000112273	chr6	22569678	22571892	+	HDGFL1	NO
ENSG00000114349	chr3	50229045	50233949	+	GNAT1	NO
ENSG00000114487	chr3	108677086	108836989	-	MORC1	YES
ENSG00000116726	chr1	12834984	12838049	+	PRAMEF12	NO
ENSG00000117148	chr1	18081808	18153558	+	ACTL8	YES
ENSG00000120210	chr9	5131979	5185668	-	INSL6	NO
ENSG00000120498	chrX	69748790	70128581	-	TEX11	NO
ENSG00000121446	chr1	182419256	182529734	+	RGSL1	NO
ENSG00000123569	chrX	103265719	103268259	-	H2BFWT	NO
ENSG00000123576	chrX	103494719	103499614	-	ESX1	NO
ENSG00000123584	chrX	148663309	148669116	-	MAGEA9B	YES
ENSG00000124092	chr20	56071035	56100708	-	CTCFL	YES
ENSG00000124260	chrX	151301782	151307033	-	MAGEA10	YES
ENSG00000124678	chr6	35085848	35116387	-	TCP11	NO
ENSG00000125207	chr12	130822432	130857182	+	PIWIL1	YES
ENSG00000125788	chr20	123010	126392	+	DEFB126	NO
ENSG00000126752	chrX	48114752	48126879	+	SSX1	YES
ENSG00000129221	chr17	6297013	6338519	-	AIPL1	NO
ENSG00000131050	chr20	31749574	31769218	+	BPIFA2	NO
ENSG00000131126	chr19	43905648	43922763	+	TEX101	YES
ENSG00000131738	chr17	39519746	39526052	-	KRT33B	NO
ENSG00000131914	chr1	26737269	26756213	+	LIN28A	NO
ENSG00000132446	chrX	31089360	31090170	-	FTHL17	YES
ENSG00000133980	chr14	74769772	74826711	+	VRTN	NO
ENSG00000134588	chrX	132158659	132231137	-	USP26	NO
ENSG00000134640	chr11	92702886	92718232	+	MTNR1B	NO

ENSG00000135248	chr7	128349115	128371797	+	FAM71F1	NO
ENSG00000137674	chr11	102447566	102496063	-	MMP20	NO
ENSG00000138684	chr4	123533783	123542224	-	IL21	NO
ENSG00000138813	chr4	100432161	100463460	+	C4orf17	NO
ENSG00000139151	chr12	18836103	18890991	-	PLCZ1	NO
ENSG00000139445	chr12	109715784	109747025	-	FOXN4	NO
ENSG00000140623	chr16	4827670	4838522	-	43355	NO
ENSG00000140798	chr16	48116884	48189929	-	ABCC12	NO
ENSG00000141096	chr16	68009566	68014732	-	DPEP3	NO
ENSG00000141255	chr17	3343313	3417146	-	SPATA22	NO
ENSG00000141316	chr17	31297394	31324895	+	SPACA3	YES
ENSG00000142025	chr19	42348806	42356401	+	DMRTC2	NO
ENSG00000142513	chr19	51293672	51298481	+	ACPT	NO
ENSG00000142698	chr1	34632484	34684732	+	C1orf94	NO
ENSG00000143006	chr1	53925072	53933161	+	DMRTB1	NO
ENSG00000143032	chr1	91177096	91182794	-	BARHL2	NO
ENSG00000143194	chr1	166958346	166991451	+	MAEL	YES
ENSG00000144015	chr2	96257766	96265526	+	TRIM43	NO
ENSG00000145309	chr4	71200683	71202833	+	CABS1	NO
ENSG00000146276	chr6	89887220	89940997	-	GABRR1	NO
ENSG00000146453	chr6	160221298	160241736	+	PNLDC1	NO
ENSG00000147183	chrX	88002226	88009786	+	CPXCR1	YES
ENSG00000147381	chrX	151080981	151093642	+	MAGEA4	YES
ENSG00000151005	chr4	164392257	164395047	-	TKTL2	NO
ENSG00000151033	chr10	30900718	30918691	-	LYZL2	NO
ENSG00000151360	chr2	3705785	3750261	+	ALLC	NO
ENSG00000151615	chr4	147560045	147563626	+	POU4F2	NO
ENSG00000151962	chr4	155702365	155749965	+	RBM46	YES
ENSG00000152086	chr2	130949318	130956034	-	TUBA3E	NO
ENSG00000152208	chr4	93225550	94695707	+	GRID2	NO
ENSG00000152463	chr10	15074226	15115851	+	OLAH	NO
ENSG00000152670	chr5	55033845	55112985	+	DDX4	NO
ENSG00000153779	chrX	89176881	89177882	+	TGIF2LX	NO
ENSG00000154143	chr11	124481386	124490252	+	PANX3	NO
ENSG00000154438	chr7	117003276	117068177	-	ASZ1	NO
ENSG00000154611	chr18	23713816	23773319	+	PSMA8	NO
ENSG00000154997	chr7	55861237	55930482	-	43357	NO
ENSG00000155495	chrX	140991680	140997174	+	MAGEC1	YES
ENSG00000155875	chr9	18927656	19033251	-	FAM154A	NO
ENSG00000156009	chrX	149009941	149014609	+	MAGEA8	YES
ENSG00000156269	chr4	80146848	80247204	-	NAA11	NO
ENSG00000157060	chr1	182869000	182922660	-	SHCBP1L	NO
ENSG00000157884	chr2	26804070	26864236	-	CIB4	NO
ENSG00000158639	chrX	55246788	55250541	+	PAGE5	YES
ENSG00000159224	chr17	47035916	47045958	-	GIP	NO
ENSG00000160505	chr19	56347944	56393220	+	NLRP4	YES
ENSG00000161040	chr7	102453308	102715286	-	FBXL13	NO
ENSG00000161609	chr19	49891475	49921251	+	CCDC155	NO

ENSG00000161652	chr19	50655805	50666452	-	IZUMO2	NO
ENSG00000162039	chr16	1883984	1934295	-	MEIOB	NO
ENSG00000162624	chr1	75594119	75627218	+	LHX8	NO
ENSG00000162753	chr1	173469603	173572233	-	SLC9C2	NO
ENSG00000162771	chr1	212797789	212800120	+	FAM71A	NO
ENSG00000163206	chr1	152850793	152857523	+	SMCP	NO
ENSG00000163263	chr1	154171848	154178809	-	C1orf189	NO
ENSG00000163424	chr3	118864997	118878889	+	C3orf30	NO
ENSG00000163440	chr4	56422692	56458379	-	PDCL2	NO
ENSG00000163530	chr3	109012635	109035364	-	DPPA2	YES
ENSG00000163793	chr2	27498289	27504367	+	DNAJC5G	NO
ENSG00000164256	chr5	23507264	23528706	+	PRDM9	NO
ENSG00000164299	chr5	79615444	79617661	+	SPZ1	NO
ENSG00000164744	chr7	48026745	48068716	-	SUN3	NO
ENSG00000164900	chr7	150845676	150871832	-	GBX1	NO
ENSG00000165131	chr7	142636440	142637955	+	C7orf34	NO
ENSG00000165496	chr14	47120222	47121028	-	RPL10L	NO
ENSG00000165583	chrX	48045656	48056199	-	SSX5	YES
ENSG00000165584	chrX	48205863	48216142	-	SSX3	YES
ENSG00000165643	chr9	138585253	138591374	-	SOHLH1	NO
ENSG00000165863	chr10	118423207	118429775	-	C10orf82	NO
ENSG00000165972	chr12	96260826	96336752	-	CCDC38	NO
ENSG00000166049	chrX	150732094	150845211	+	PASD1	YES
ENSG00000166069	chr15	38214140	38259925	+	TMCO5A	NO
ENSG00000166118	chr11	133710526	133715433	-	SPATA19	YES
ENSG00000166800	chr11	18477371	18501147	+	LDHAL6A	NO
ENSG00000166926	chr11	60102304	60164069	+	MS4A6E	NO
ENSG00000168070	chr11	64704989	64739557	-	C11orf85	NO
ENSG00000168454	chr18	9885763	9889272	+	TXNDC2	NO
ENSG00000168757	chrY	6114264	6117060	+	TSPY2	YES
ENSG00000168930	chr11	89530823	89541743	-	TRIM49	NO
ENSG00000169059	chrX	6451659	6453159	-	VCX3A	NO
ENSG00000169551	chrX	134290461	134305322	-	CXorf48	YES
ENSG00000170279	chr7	148287657	148312952	+	C7orf33	NO
ENSG00000170486	chr12	52979373	52995322	-	KRT72	NO
ENSG00000170516	chr4	46736844	46911252	-	COX7B2	NO
ENSG00000170788	chr10	82095861	82116511	-	DYDC1	NO
ENSG00000171102	chr9	136080664	136084630	-	OBP2B	NO
ENSG00000171209	chr4	71108305	71117145	+	CSN3	NO
ENSG00000171405	chrX	52841228	52847325	+	XAGE5	YES
ENSG00000171487	chr19	56511092	56573179	+	NLRP5	NO
ENSG00000171772	chr10	135367404	135382876	-	SYCE1	YES
ENSG00000171804	chr19	38375463	38397317	-	WDR87	NO
ENSG00000171956	chr15	60296421	60353929	+	FOXB1	NO
ENSG00000172967	chr22	17264302	17302589	-	XKR3	NO
ENSG00000173809	chr19	33210659	33320483	+	TDRD12	NO
ENSG00000174016	chrX	79591003	79700810	+	FAM46D	YES
ENSG00000175018	chr10	127265091	127371713	-	TEX36	NO

ENSG00000175294	chr11	65784223	65793988	-	CATSPER1	NO
ENSG00000175664	chr13	31506840	31549639	+	TEX26	NO
ENSG00000176294	chr14	20187174	20296531	+	OR4N2	NO
ENSG00000176566	chr8	88882973	88886296	-	DCAF4L2	NO
ENSG00000176635	chr22	30476163	30573064	+	HORMAD2	YES
ENSG00000176679	chrY	3447082	3448082	+	TGIF2LY	NO
ENSG00000176746	chrX	26210557	26213763	+	MAGEB6	YES
ENSG00000177138	chrX	8992273	9132680	-	FAM9B	NO
ENSG00000177414	chr1	64669310	64733051	+	UBE2U	NO
ENSG00000177627	chr12	48876286	48890295	+	C12orf54	NO
ENSG00000177689	chrX	27826107	27841131	+	MAGEB10	NO
ENSG00000177938	chr12	18891045	18892121	+	CAPZA3	NO
ENSG00000177992	chr9	90497741	90503814	+	SPATA31E1	NO
ENSG00000177994	chr2	54557171	54610879	+	C2orf73	NO
ENSG00000178125	chr8	67876334	67968839	-	PPP1R42	NO
ENSG00000178403	chr4	113434672	113437328	-	NEUROG2	NO
ENSG00000178645	chr10	50887697	50918307	+	C10orf53	NO
ENSG00000179046	chr4	189012427	189030757	-	TRIML2	NO
ENSG00000179059	chr4	188916925	188926204	+	ZFP42	NO
ENSG00000179709	chr19	56459198	56499995	+	NLRP8	NO
ENSG00000179873	chr19	56296770	56348166	-	NLRP11	NO
ENSG00000180043	chr19	55866276	55874628	-	FAM71E2	NO
ENSG00000180083	chr20	44277202	44298909	-	WFDC11	NO
ENSG00000180869	chr1	85093913	85100703	-	C1orf180	NO
ENSG00000181433	chrX	134971374	134995220	+	SAGE1	YES
ENSG00000181562	chr14	21214051	21216539	+	EDDM3A	NO
ENSG00000181781	chr19	463346	474983	-	ODF3L2	NO
ENSG00000181786	chr19	8807751	8809172	-	ACTL9	NO
ENSG00000182111	chr7	57509883	57533252	+	ZNF716	NO
ENSG00000182459	chr17	80317123	80321652	+	TEX19	NO
ENSG00000182583	chrX	7810303	7812184	+	VCX	NO
ENSG00000183324	chr15	73735499	73852355	+	C15orf60	YES
ENSG00000183434	chrX	132350697	132352376	-	TFDP3	YES
ENSG00000183559	chr10	124457225	124459338	-	C10orf120	NO
ENSG00000183629	chr15	28764757	28778160	-	GOLGA8G	NO
ENSG00000183654	chr5	16067248	16180871	-	43170	NO
ENSG00000183706	chr15	22382382	22383507	+	OR4N4	NO
ENSG00000184029	chr21	39323728	39493454	-	DSCR4	NO
ENSG00000184033	chrX	153845865	153847533	-	CTAG1B	YES
ENSG00000184478	chr11	5968570	5969591	+	OR56A3	NO
ENSG00000184507	chr15	34635516	34649938	+	NUTM1	NO
ENSG00000184571	chr22	25115001	25170687	-	PIWIL3	NO
ENSG00000184735	chrX	23018087	23020204	+	DDX53	YES
ENSG00000185177	chr7	57187321	57207571	-	ZNF479	NO
ENSG00000185247	chrX	148769894	148798926	+	MAGEA11	YES
ENSG00000185775	chr9	43624507	43630730	-	SPATA31A6	NO
ENSG00000185823	chr15	24920541	24928593	+	NPAP1	NO

ENSG00000186075	chr17	38024417	38034149	+	ZPBP2	NO
ENSG00000186086	chr1	108918421	109013624	+	NBPF6	NO
ENSG00000186453	chr2	24397938	24423718	+	FAM228A	NO
ENSG00000187268	chrX	13053737	13062801	-	FAM9C	NO
ENSG00000187516	chrX	37850070	37850569	+	CXorf27	NO
ENSG00000187533	chr4	70999333	71042516	+	C4orf40	NO
ENSG00000187537	chr14	19983559	20020272	-	POTEM	NO
ENSG00000187581	chr14	93813537	93814702	+	COX8C	NO
ENSG00000187600	chr2	46656329	46711564	+	TMEM247	NO
ENSG00000187690	chrX	51149767	51151687	+	CXorf67	NO
ENSG00000187772	chr6	105404923	105531207	+	LIN28B	NO
ENSG00000187806	chr15	72690614	72700370	+	TMEM202	NO
ENSG00000189023	chrX	35816459	35821852	+	MAGEB16	NO
ENSG00000189064	chrX	49354132	49361430	+	GAGE2A	YES
ENSG00000189299	chrX	55649833	55652621	+	FOXR2	NO
ENSG00000196119	chr11	124439893	124441037	+	OR8A1	NO
ENSG00000196242	chr1	247693434	247697141	-	OR2C3	NO
ENSG00000196427	chr1	108765963	108786689	-	NBPF4	NO
ENSG00000196431	chr22	27017928	27026636	+	CRYBA4	NO
ENSG00000196844	chr11	125646008	125648714	-	PATE2	NO
ENSG00000197123	chr7	63688852	63727309	+	ZNF679	NO
ENSG00000197181	chr8	22132810	22215076	+	PIWIL2	YES
ENSG00000197416	chr8	82437216	82443613	-	FABP12	NO
ENSG00000197826	chr4	81256874	81884910	+	C4orf22	NO
ENSG00000198028	chr19	9577183	9609283	-	ZNF560	NO
ENSG00000198033	chr13	19747910	19755992	-	TUBA3C	NO
ENSG00000198054	chr21	39493545	39560110	+	DSCR8	YES
ENSG00000198062	chr22	16256441	16287937	-	POTEH	YES
ENSG00000198173	chrX	37026432	37029739	+	FAM47C	NO
ENSG00000198573	chrX	140335596	140336629	-	SPANXC	YES
ENSG00000198681	chrX	152481522	152486115	-	MAGEA1	YES
ENSG00000198704	chr6	28471073	28495992	-	GPX6	NO
ENSG00000198765	chr1	115397424	115537991	+	SYCP1	YES
ENSG00000198798	chrX	30248553	30255607	+	MAGEB3	YES
ENSG00000198883	chrX	152157368	152162671	-	PNMA5	NO
ENSG00000198889	chrX	125683369	125686834	-	DCAF12L1	NO
ENSG00000203909	chr6	74062785	74064018	-	DPPA5	NO
ENSG00000203926	chrX	140677562	140678899	+	SPANXA2	YES
ENSG00000203963	chr1	67557848	67697536	-	C1orf141	NO
ENSG00000203989	chrX	119205848	119211707	-	RHOXF2B	NO
ENSG00000204661	chr5	179068545	179072047	-	C5orf60	NO
ENSG00000204671	chr12	122656577	122658746	-	IL31	NO
ENSG00000204961	chr5	140227048	140391929	+	PCDHA9	NO
ENSG00000205108	chr9	34723052	34729464	-	FAM205A	NO
ENSG00000205111	chr2	39402787	39456729	-	CDKL4	NO
ENSG00000205212	chr17	20739760	20799453	-	CCDC144NL	NO
ENSG00000205642	chrX	8432871	8434550	+	VCX3B	NO
ENSG00000205777	chrX	49363628	49373139	+	GAGE1	YES

ENSG00000213218	chr17	61949372	61951126	-	CSH2	NO
ENSG00000213231	chr14	96152754	96158980	+	TCL1B	NO
ENSG00000214107	chrX	30261847	30270155	+	MAGEB1	YES
ENSG00000214415	chr7	80087987	80141336	-	GNAT3	NO
ENSG00000215262	chr8	36641842	36793646	+	KCNU1	NO
ENSG00000215343	chr8	11961898	11973025	+	ZNF705D	NO
ENSG00000215568	chr22	17442826	17489112	-	GAB4	NO
ENSG00000221882	chr17	3181193	3182268	-	OR3A2	NO
ENSG00000224659	chrX	49178536	49185863	+	GAGE12J	YES
ENSG00000226372	chrX	27996110	27999566	-	DCAF8L1	NO
ENSG00000226941	chrY	24454970	24564028	+	RBMY1J	NO
ENSG00000227488	chrX	49306370	49313697	+	GAGE12D	YES
ENSG00000228927	chrY	9236030	9307357	+	TSPY3	YES
ENSG00000229937	chr7	18066405	18067486	-	PRPS1L1	NO
ENSG00000231171	chr4	178649911	178911904	+	RP11-389E17.1	NO
ENSG00000232423	chr1	12998181	13117751	-	PRAMEF6	NO
ENSG00000233803	chrY	9175073	9177893	+	TSPY4	NO
ENSG00000234068	chrX	55115441	55119275	+	PAGE2	YES
ENSG00000234414	chrY	23673258	23711212	+	RBMY1A1	NO
ENSG00000236311	chr10	102849078	102890883	-	TLX1NB	NO
ENSG00000236446	chrX	120006457	120009779	-	CT47B1	YES
ENSG00000237136	chr4	146601356	146692184	+	C4orf51	NO
ENSG00000238269	chrX	55101496	55105342	+	PAGE2B	YES
ENSG00000240021	chr1	178482212	178517734	+	TEX35	NO
ENSG00000241476	chrX	52725946	52736239	-	SSX2	YES
ENSG00000243543	chr20	44162835	44168134	-	WFDC6	NO
ENSG00000250423	chrX	118212598	118284542	-	KIAA1210	NO
ENSG00000251258	chr6	112668532	112672498	+	RFPL4B	NO
ENSG00000255181	chr8	144788864	144790279	-	CCDC166	NO
ENSG00000258713	chr20	2795633	2796479	+	C20orf141	NO
ENSG00000258992	chrY	9236076	9307357	+	TSPY1	NO
ENSG00000261509	chr16	33261515	33264716	+	TP53TG3B	NO

Table S2. Information of primers for Sequenom MassARRAY iPLEX.

Variants	Sequence (5'-3')
rs12645087	F: ACGTTGGATGAGGCTCTGGCTTATGGTTGA<br>R: ACGTTGGATGCTGACCTTCACGTGTTTTAC<br>E: ccCGTGTTTTACATAATAAAATGCC
rs145033304	F: ACGTTGGATGGCCGCTATAAACTAGAATGG<br>R: ACGTTGGATGACACTGACTTCCACAATGGC<br>E: CACTGTTGGTGGGAC
rs17135666	F: ACGTTGGATGATGGTCTTACTGATCAGAGG<br>R: ACGTTGGATGATCTGCACATCACATCTCCC<br>E: tggtCAACCCAAATTTTCCCT
rs175150	F: ACGTTGGATGCTTGGATTTTAGTTGGCAAAG<br>R: ACGTTGGATGAGTTTATTGCGCAAGTGAGG<br>E: ctcgTCTTCATTACAATTCATACCTAG
rs3123484	F: ACGTTGGATGAGACACAAGTAGGTTAACAG<br>R: ACGTTGGATGTGTCTCTAGCAACAATTGTG<br>E: AGAGCTGGGGTGGCTA
rs402969	F: ACGTTGGATGCTAGACCTAAGAAGCAAGGG<br>R: ACGTTGGATGTGCATTGCACCGATTGGAAG<br>E: gggatCTCCCTTGATTCAGAATGAATG
rs60813831	F: ACGTTGGATGGGAGACCCACTAAATTGCTG<br>R: ACGTTGGATGCCTGTCTGGACTAAATCCTG<br>E: ctacATCCTGTTTTAGTCAGCAG
rs6941653	F: ACGTTGGATGGGAAATATGTGATCAGGAGG<br>R: ACGTTGGATGGTTGAGCATTTTTTGGCAGG<br>E: TTCCAATATATTTCTTTAGAAAATGT
rs7546603	F: ACGTTGGATGCTCCCCTCTTTATACACACG<br>R: ACGTTGGATGTGAGGTCCTGCACATACGG<br>E: gggagACACACGTATGAAAATACATCTA
rs77027865	F: ACGTTGGATGAGAGGCAGAGGGAAGCATAG<br>R: ACGTTGGATGGACATGGCAGTGATTTTTGAC<br>E: atGCTGGAGAAAGGACAG
rs79461429	F: ACGTTGGATGCCTGCTTATATTTCCATTGC<br>R: ACGTTGGATGACAAGATCGATCTGCATCTG<br>E: ACCTGTAAAGACACACA
rs79727953	F: ACGTTGGATGAGTGATGCGCTGAGTTCTTC<br>R: ACGTTGGATGACAAAGGTTATCCTGGGTGG<br>E：gacgGGGTGGGCCTGACCTAATCAA
rs8139987	F: ACGTTGGATGGAGAAGTCTCTAATCTCCTC<br>R: ACGTTGGATGACTTGATGTGAATGGATGGG<br>E: ggCAGTTTTTGGAAACATGC
rs9478496	F: ACGTTGGATGTGGACTTCCACTTCCAAATG

	R: ACGTTGGATGCAGTCCAAACCTTTTGCTGA<br>E: TTTTAGCAGTTATATGATGAGAC
rs6851719	F: ACGTTGGATGGACAGTAACACAGTGGTTTC<br>R: ACGTTGGATGGACTGCATTCATCATGTGGC<br>E: ATTTGCTTTTAATCACTGTTC
rs75932085<br>(rs144031443)	F: ACGTTGGATGGGCATACAATCAGACAAGAC<br>R: ACGTTGGATGACCAGTCATCAGGAATAAGG<br>E: GCTGGTTTACTGGGTTA
rs4726004<br>(rs150492976)	F: ACGTTGGATGTTCTGGGACATGAGTGCATC<br>R: ACGTTGGATGTGGTGACTCCTGAAACTCTG<br>E: ATGAGTGCATCTTTGGGTA

Note: primers for rs144031443 and rs150492976 were not successfully designed due to too much "ATATAT" in the sequences. These two variants were replaced by rs75932085 (r^2 = 0.66) and rs4726004 (r^2 = 1), respectively.

Table S3. Interaction analysis between our identified SNPs (rs6941653 and rs402969) and smoking.

Stage	Smoke	rs6941653 Genotype									P_interaction
		TT			TC			CC			
		Cases/Controls	OR (95%CI)	*P*	Cases/Controls	OR (95%CI)	*P*	Cases/Controls	OR (95%CI)	*P*	
**NJMU GWAS**	NO	497/1111	1.00	Reference	286/588	1.10(0.92-1.31)	0.292	42/69	1.34(0.90-2.00)	0.155	0.287
	Yes	863/835	3.17(2.67-3.77)	6.58E-39	566/420	4.13(3.41-5.01)	1.51E-47	77/54	4.27(2.93-6.22)	3.81E-14	
**Validation**	NO	353/364	1.00	Reference	186/166	1.15(0.89-1.49)	0.272	26/22	1.22(0.68-2.20)	0.501	0.644
	Yes	262/275	1.07(0.83-1.38)	0.597	160/124	1.45(1.07-1.97)	0.016	20/18	1.25(0.64-2.43)	0.513	
**Combined**	NO	850/1475	1.00	Reference	472/754	1.09(0.95-1.26)	0.227	68/91	1.29(0.93-1.79)	0.129	0.172
	Yes	1125/1110	2.20(1.91-2.53)	3.41E-28	726/544	2.89(2.47-3.39)	1.80E-39	97/72	2.88(2.08-3.98)	1.69E-10	
**Stage**	**Smoke**	**rs402969 Genotype**									***P*_interaction**
		**CC**			**CT**			**TT**			
		**Cases/Controls**	**OR (95%CI)**	***P***	**Cases/Controls**	**OR (95%CI)**	***P***	**Cases/Controls**	**OR (95%CI)**	***P***	
**NJMU GWAS**	NO	539/1188	1.00	Reference	249/521	1.07(0.89-1.28)	0.498	37/59	1.33(0.87-2.04)	0.189	0.341
	Yes	983/913	3.25(2.75-3.85)	3.89E-43	457/356	3.85(3.16-4.69)	6.61E-41	66/40	5.00(3.29-7.59)	4.47E-14	
**Validation**	NO	348/358	1.00	Reference	191/184	1.06(0.83-1.37)	0.628	26/20	1.33(0.73-2.43)	0.355	0.365
	Yes	281/290	1.07(0.83-1.38)	0.614	143/118	1.33(1.00-1.82)	0.049	14/11	1.39(0.62-3.14)	0.423	
**Combined**	NO	887/1546	1.00	Reference	440/705	1.09(0.95-1.27)	0.226	63/79	1.36(0.97-1.92)	0.077	0.222
	Yes	1264/1203	2.28(1.99-2.61)	3.54E-32	600/474	2.73(2.32-3.22)	2.07E-33	80/51	3.39(2.35-4.91)	7.94E-11	

Table S4. Interaction analysis between risk variant rs6941653 and SNP rs402969.

Stage	rs6941653 Genotype	rs402969 Genotype									P_interaction
		CC			CT			TT			
		Cases/Controls	OR (95%CI)	*P*	Cases/Controls	OR (95%CI)	*P*	Cases/Controls	OR (95%CI)	*P*	
**NJMU GWAS**	TT	894/1357	1.00	Reference	413/532	1.18(1.01-1.38)	0.039	53/57	1.37(0.93-2.04)	0.116	0.787
	TC	548/661	1.25(1.08-1.44)	0.003	262/307	1.32(1.09-1.59)	0.005	42/40	1.64(1.04-2.58)	0.033	
	CC	80/83	1.37(0.98-1.90)	0.062	31/38	1.31(0.80-2.16)	0.283	8/2	5.77(1.19-27.94)	0.029	
**Validation**	TT	365/424	1.00	Reference	210/191	1.28(1.02-1.63)	0.036	26/18	1.66(0.89-3.08)	0.108	0.094
	TC	233/178	1.52(1.19-1.93)	6.79E-04	98/93	1.24(0.90-1.70)	0.185	9/11	0.96(0.39-2.34)	0.923	
	CC	24/24	1.17(0.65-2.09)	0.904	16/14	1.32(0.63-2.74)	0.461	5/2	2.92(0.56-15.14)	0.203	
**Combined**	TT	1259/1781	1.00	Reference	623/723	1.23(1.07-1.40)	0.002	79/75	1.48(1.07-2.06)	0.019	0.276
	TC	781/839	1.30(1.15-1.47)	3.28E-05	360/400	1.30(1.10-1.53)	0.002	51/51	1.45(0.97-2.17)	0.068	
	CC	104/107	1.32(1.00-1.76)	0.054	47/52	1.31(0.87-1.97)	0.195	13/4	4.62(1.49-14.38)	0.008	

Age, gender, smoking and principal components were adjusted when appropriate.

Table S5. Pathway enrichment and GO analysis.

Gene	Category	Term	Count	%	*P*	Fold [a]	Bonferroni [b]
OPRM1	KEGG_PATHWAY	Olfactory transduction	194	46.6	2.10E-214	14.0	2.00E-212
	GO_MF	olfactory receptor activity	196	47.1	8.00E-243	23.9	1.50E-240
	GO_MF	G-protein coupled receptor activity	210	50.5	1.00E-215	15.6	2.00E-213
	GO_MF	odorant binding	47	11.3	4.10E-55	26.9	7.90E-53
	GO_MF	transmembrane signaling receptor activity	48	11.5	1.20E-36	11.8	2.40E-34
	GO_MF	type I interferon receptor binding	8	1.9	1.40E-08	24.7	2.60E-06
	GO_MF	trace-amine receptor activity	4	1.0	1.30E-04	35.1	2.50E-02
	GO_MF	adrenergic receptor activity	4	1.0	2.20E-04	30.1	4.20E-02
	GO_CC	plasma membrane	227	54.6	4.90E-68	2.9	5.50E-66
	GO_CC	integral component of membrane	240	57.7	5.80E-59	2.5	6.50E-57
	GO_CC	keratin filament	12	2.9	2.60E-06	6.4	2.90E-04
	GO_BP	detection of chemical stimulus involved in sensory perception of smell	196	47.1	5.70E-246	24.5	2.80E-243
	GO_BP	G-protein coupled receptor signaling pathway	204	49.0	2.10E-183	12.1	1.00E-180
	GO_BP	sensory perception of smell	57	13.7	1.00E-56	19.0	4.90E-54
	GO_BP	detection of chemical stimulus involved in sensory perception	48	11.5	2.90E-56	26.8	1.50E-53
	GO_BP	natural killer cell activation involved in immune response	8	1.9	3.20E-08	22.4	1.60E-05
	GO_BP	positive regulation of peptidyl-serine phosphorylation of STAT protein	8	1.9	3.20E-08	22.4	1.60E-05
	GO_BP	T cell activation involved in immune response	8	1.9	1.00E-07	19.3	5.00E-05
	GO_BP	regulation of type I interferon-mediated signaling pathway	8	1.9	3.70E-07	16.4	1.80E-04
	GO_BP	B cell proliferation	8	1.9	1.70E-06	13.3	8.50E-04
	GO_BP	response to exogenous dsRNA	8	1.9	2.70E-06	12.5	1.30E-03
	GO_BP	B cell differentiation	9	2.2	3.10E-05	7.2	1.50E-02
	GO_BP	adenylate cyclase-activating serotonin receptor signaling pathway	5	1.2	7.60E-05	20.4	3.70E-02
	GO_BP	humoral immune response	8	1.9	9.00E-05	7.5	4.40E-02
NLRP8	KEGG_PATHWAY	Olfactory transduction	176	16.1	3.10E-110	6.7	7.60E-108

	GO_MF	olfactory receptor activity	177	16.2	1.00E-111	7.7	9.10E-109
	GO_MF	G-protein coupled receptor activity	193	17.7	3.30E-85	5.1	2.90E-82
	GO_MF	odorant binding	45	4.1	1.40E-31	9.1	1.20E-28
	GO_MF	transmembrane signaling receptor activity	50	4.6	1.80E-18	4.4	1.50E-15
	GO_MF	type I interferon receptor binding	8	0.7	1.50E-05	8.8	1.30E-02
	GO_MF	structural molecule activity	31	2.8	2.50E-05	2.3	2.10E-02
	GO_CC	plasma membrane	379	34.7	1.30E-33	1.7	5.80E-31
	GO_CC	integral component of membrane	404	37.0	3.50E-21	1.5	1.60E-18
	GO_CC	extracellular region	127	11.6	3.30E-06	1.5	1.50E-03
	GO_BP	detection of chemical stimulus involved in sensory perception of smell	177	16.2	1.50E-113	7.8	4.70E-110
	GO_BP	G-protein coupled receptor signaling pathway	200	18.3	1.80E-72	4.2	5.70E-69
	GO_BP	sensory perception of smell	57	5.2	1.80E-31	6.7	5.70E-28
	GO_BP	detection of chemical stimulus involved in sensory perception	45	4.1	5.70E-31	8.9	1.80E-27
	GO_BP	keratinocyte differentiation	17	1.6	1.90E-06	4.2	6.10E-03

[a] Fold enrichment in each pathway or GO process.

[b] *P* values after taking Bonferroni correction.

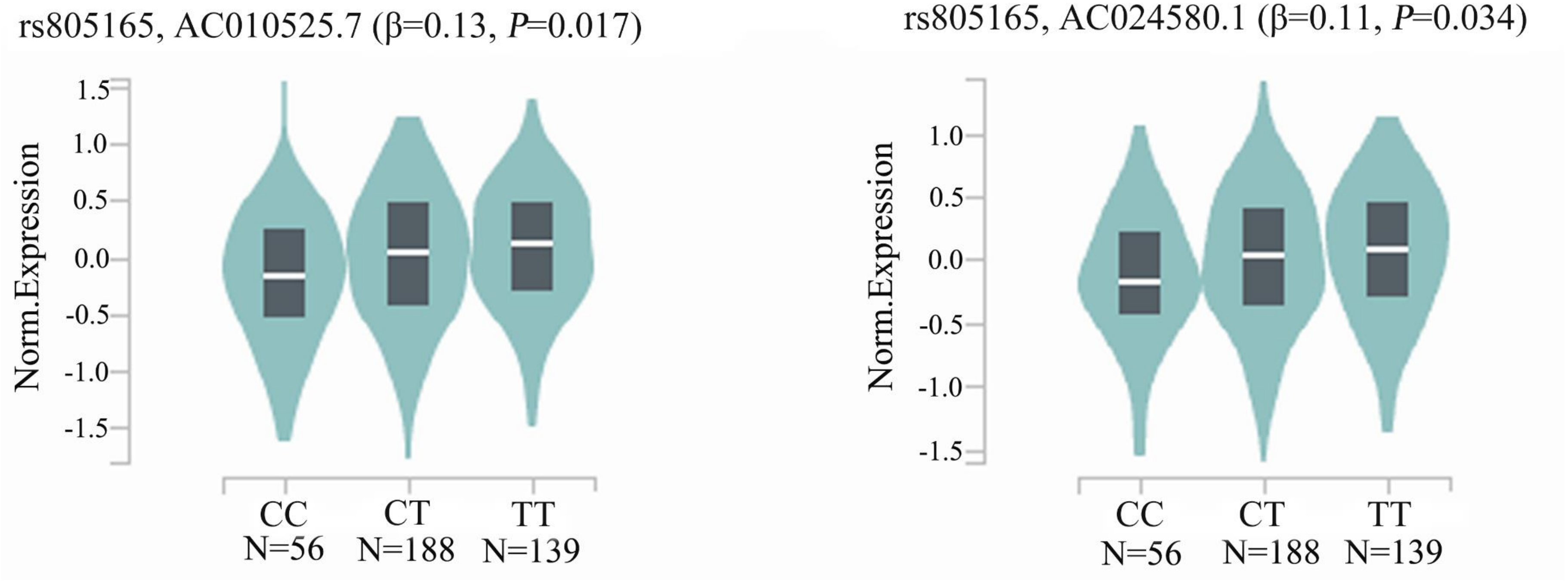

Supplementary Figure 1. Rs805165 was significantly associated with the expression of AC010525.7 and AC024580.1 in lung tissues based on GTEx v7 database.

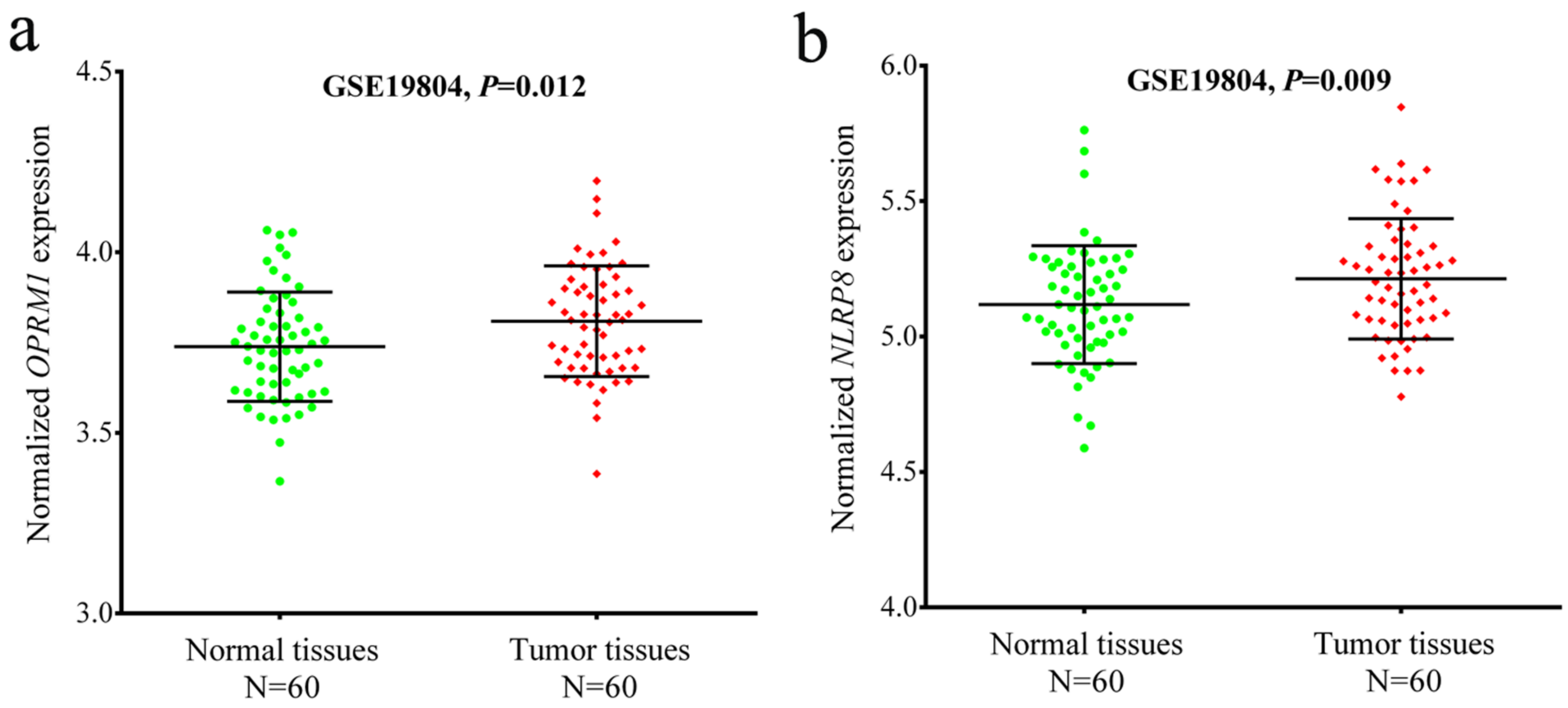

Supplementary Figure 2. Expression of *OPRM1* and *NLRP8* in 60 paired lung tumor tissues and adjacent normal tissues based on GSE19804.
